# Charge tunable thin-film composite membranes by gamma-ray triggered surface polymerization

**DOI:** 10.1038/s41598-017-04900-5

**Published:** 2017-06-30

**Authors:** Rackel Reis, Mikel C. Duke, Blaise L. Tardy, Daniel Oldfield, Raymond R. Dagastine, John D. Orbell, Ludovic F. Dumée

**Affiliations:** 1 Institute for Sustainability for Innovation, College of Engineering and Science, Victoria University, Melbourne, VIC 3030 Australia; 2 Deakin University, Institute for Frontier Materials, Geelong, VIC 3216 Australia; 3 RMIT, School of Science, Applied Science, Melbourne, VIC 3000 Australia; 4 Department of Biomolecular and Chemical Engineering, The University of Melbourne, Melbourne, VIC 3010 Australia; 5 School of Applied Sciences, RMIT University, Melbourne, VIC 3030 Australia

## Abstract

Thin-film composite poly(amide) (PA) membranes have greatly diversified water supplies and food products. However, users would benefit from a control of the electrostatic interactions between the liquid and the net surface charge interface in order to benefit wider application. The ionic selectivity of the 100 nm PA semi-permeable layer is significantly affected by the pH of the solution. In this work, for the first time, a convenient route is presented to configure the surface charge of PA membranes by gamma ray induced surface grafting. This rapid and up-scalable method offers a versatile route for surface grafting by adjusting the irradiation total dose and the monomer concentration. Specifically, thin coatings obtained at low irradiation doses between 1 and 10 kGy and at low monomer concentration of 1 v/v% in methanol/water (1:1) solutions, dramatically altered the net surface charge of the pristine membranes from −25 mV to +45 mV, whilst the isoelectric point of the materials shifted from pH 3 to pH 7. This modification resulted in an improved water flux by over 55%, from 45.9 to up 70 L.m^−2^.h^−1^, whilst NaCl rejection was found to drop by only 1% compared to pristine membranes.

## Introduction

The nature and distribution of chemical functional groups across the surface of thin-film composite (TFC) membranes is critical to the tuning of the selectivity of membrane materials in relation to seawater desalination and other applications including foods processing^1^. The excellent salt and organic rejection capability of poly(amide) (PA) TFC membranes is due to a combination of the material’s free volume, which corresponds to the spaces between the macromolecular chains, and to the nature of the electrostatic repulsions at the liquid/membrane interface, *i.e*. the Donnan Layer^2^. Such electrostatic interactions are influenced by the polar moieties that are present on the surface of the membrane material^3^. The pH dependent Donnan Layer effectively acts as a barrier to ionic species, with rejection characteristics being influenced by the charge, hydration, valence and size of ions^3^.

The chemical composition of the active PA layer, prepared by interfacial polymerization (IP) of two non-miscible monomers^4^, is rich in carboxylate and aromatic amide groups resulting in an amphiphilic character, although being largely negatively charged overall. The IP process leads to the formation of a rough surface, with protuberances on the order of 100 nm. This roughness arises from surface tension and nano-scale flow pattern effects between the two non-miscible phases upon polymerization. The resultant morphology facilitates water transport across the membrane by offering a high surface area to volume ratio and also by the creation of nano-voids or free volume across the thin layer^5^. The negative surface charge will favor the adsorption of positively charged proteins, cationic surfactants, solvent molecules or ions due to preferential electrostatic interactions, which may chemisorb onto the surface. Such adsorption mechanisms, which contribute to fouling, depend on the nature of the contaminants, and lead to permeation decline and to pH dependent salt rejection capabilities^6^. Routes to control the degree of positively charged functional groups across the exposed surface of TFC membranes are therefore needed to fine-tune membrane selectivity, reduce the adverse effects of fouling and to generate a more versatile generation of desalination membranes^7^. Irradiation-induced grafting is a viable concept to tune surface charge, with the potential of creating higher performance membrane materials. Other conventional chemical routes require several steps and significant amount of chemicals making scale-up membrane production difficult^8, 9^.

Here, we demonstrate this innovative approach on commercial flat sheet TFC membranes, across a range of irradiation doses and environmental conditions. The monomer 1-vinylimidazole (VIM) was used for the gamma irradiation-facilitated surface grafting of amine groups in order to influence the charge characteristics on the material. VIM is a promising monomer candidate that contains reactive vinyl and imidazole moieties which may covalently react with PA materials^10^. A systematic investigation of the resultant amine coverage and surface texture properties was performed and critically correlated to membrane performance. This study demonstrates for the first time the feasibility and potential of irradiation-induced grafting onto functional thin-film membrane materials as a more sustainable and cost-effective approach than other methods with a potential for specific selectivity and tailored performance.

## Materials and Methods

### Monomer solution and surface grafting

Radiation grafting was performed at the Gamma Technology Research Irradiator (GATRI) facilities at the Australian Nuclear Science and Technology Organization (ANSTO). BW30 TFC membranes, purchased from Dow Filmtec Corp. (IMCD Limited Australia), were immersed separately in three different concentrations (1, 10 and 35 v/v %) of 1-vinylimidazole (VIM) (Sigma Aldrich ≥ 99%) 50 v/v% of methanol/water solutions^11^. The grafting process was performed for total irradiation doses of 1, 10 and 100 kGy, corresponding to 0.4, 4 and 42 h of irradiation respectively. More detail is provided in the supplementary materials, including the mass gain equation^12^. Upon irradiation, the samples were thoroughly and sequentially rinsed with water and methanol for 3 times. This rinsing step ensured that no remaining unreacted monomer materials remained present across the surface of the membranes. In addition, the membranes were stored in SMBS and re-rinsed with water prior to both characterization and permeation testing. A series of pristine membranes were separated in three groups for characterization and performance testing. Namely, i) pristine membrane - as supplied; ii) pristine membrane - exposed to MeOH/water and also irradiated with the same doses as for the grafting and iii) pristine membrane not irradiated, but immersed in VIM solution for 42 h, corresponding to an equivalent irradiation at 100 kGy.

### Characterization techniques

The morphology of the modified surfaces was evaluated by scanning electron microscopy (SEM) and atomic force microscope (AFM). The SEM images were acquired on a FEI Quanta dual beam Gallium (Ga) Focused Ion Beam (FIB) microscope collected under 5 kV of accelerating voltage and for a working distance of 10 mm after thin gold sputter-coating (5 nm). AFM tests were performed on a Cypher (Asylum Research) using a Herzian TS-150 active vibration table and an ARC2 SPM controller. The manufacturer nominal value for the resonance frequency and spring constant of the cantilever were 300 kHz (±100 kHz) and 40 N/m, respectively. X-ray photoelectron spectroscopy (XPS) was utilized for surface and interface characterization. XPS was performed using a XPS Spectrometer Thermo K-alpha and quantitative elemental composition of the modified PA was provided for a surface depth of 1–5 nm. The technique was able to detect elements with a detection limit of 0.1% of the bulk material. An Al Kα (1,486.6 eV) x-ray source was used as the excitation source, and the anode was maintained at 250 W, 10 kV, and 27 mA at a chamber pressure of 2.67 10^−8^ Pa with a beam spot size of 400 μm x 400 μm. The peak position was calibrated using the C1s peak at 284.6 eV. Attenuated Total Reflection-Fourier Transform Infra-red Spectroscopy (ATR-FTIR) analysis was performed to investigate the chemical profiles induced by the gamma ray grafting. The analysis of the bands was performed using a Perkin Elmer Frontier FTIR spectrophotometer with a KBr beam splitter. All spectral areas were collected across a wavenumber range of 4000–600 cm^−1^, and final data averaged over 8 spectra at a resolution of 4 cm^−1^. Analysis was performed with OPUS 7.2 software from the Bruker Corporation. Streaming potential tests were performed to measure the surface charge of the membranes after grafting with a Surpass Anton Paar Electro Kinetic Analyser (EKA) utilizing Visiolab software (version 2.2) following a previously described procedure^13^. The membranes were cut and taped onto a 20 mm × 10 mm adjustable gap cell. The streaming channel dimension was approximately 0.1 mm. The pH electrodes (Schott Instruments) were used for measuring of zeta potential at pressure of 400 mbar. A 1 mM NaCl solution was used and 0.1 M HCl and NaOH were used for pH adjustment. An average value of the zeta potential was calculated based on four repeat measurements obtained from both directions of flow in the cell. Water contact angle measurements were acquired on a Biolin Scientific goniometer to evaluate macroscopic variations of both chemical and morphological characteristics of the PA surface layers. Prior to contact angle measurements, the membranes were dried in air overnight. The test involved adding 4 μL of de-ionized water drops in three different spots across membrane surfaces. Images were acquired 5 s after drop impact and contact angles were calculated by fitting the outline of the drops image to the Young-Laplace equation using the Biolin software. The mass gain assessed the resultant degree of polymerization on the grafted material and was carried out as previously reported^14^.

### Membranes desalination performance tests

The membranes used for permeation tests were thoroughly rinsed with DI water after irradiation to remove un-reacted materials. The circulating feed stream contained 2,000 ppm of sodium chloride (NaCl) at 25 °C, pH 6 and 15 bar following a previously reported procedure^7^. Extra details are provided in the supplementary materials.

## Results and Discussion

### Performance of the irradiated membranes

**Figure 1:**
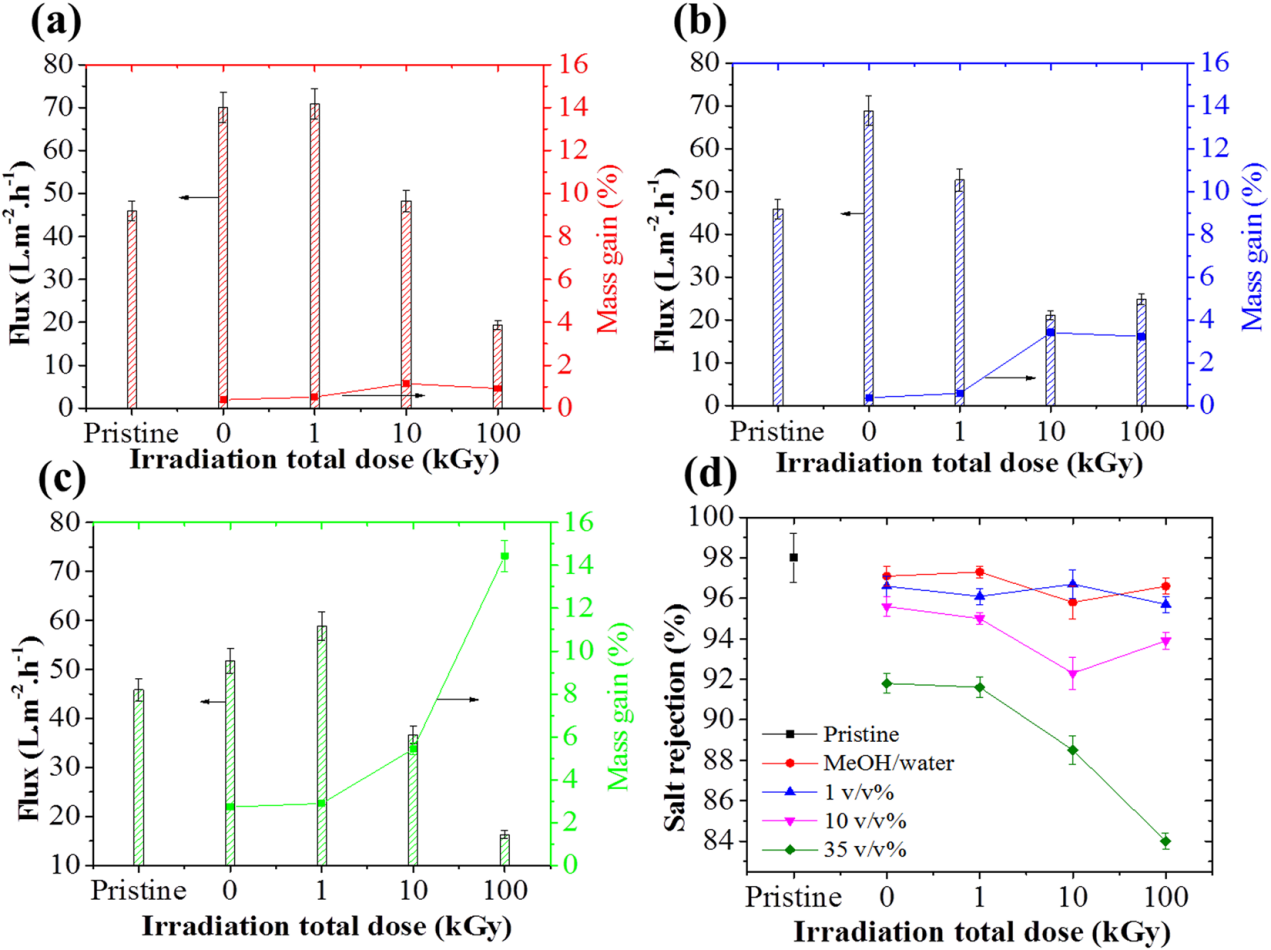
Flux and salt rejection for irradiation-induced grafted membranes; (**a**) grafting with 1 v/v% of VIM concentration, (**b**) 10 v/v% of VIM concentration, (**c**) 35 v/v% of VIM concentration and (**d**) salt rejection. Cross-flow desalination test conditions: 15 bar inlet pressure and 2,000 ppm NaCl solution at 25 °C. Details about error bars are provided in the supplement material.

Figure 1 presents the membrane water flux against different irradiation doses and the material mass gain after irradiation-induced grafting. Mass gain indicates the extent of polymerization on the surface of the treated material (Eq. S1).

As seen in Fig. 1a, the flux across the membranes irradiated in the 1 v/v% solutions was found to increase by up to 56%, compared to the pristine un-irradiated membranes, reaching 70 L.m^−2^.h^−1^ at low irradiation dose, ~1.5 and 3.5 times higher than that of grafted membranes at 10 and 100 kGy, respectively. Although mass gains showed limited overall increases from 0.52 to 1.1 wt%, the flux progressively declined as the mass gain increased with the irradiation total dose.

The flux enhancement effect suggests that the MeOH/water solvent molecules not only can facilitate the VIM diffusion across the membrane layers but also causes alterations in the free volume. This is consistent with a previous study on a PA hollow fiber forward osmosis (FO) membrane treated with N-methyl pyrrolidone as a function of the impact of oxidation resistance post-treatment^8^. The water flux of PA FO membranes with oxidation treatment increased 6 to 8 times, but the salt retention had little to no change. On the other hand, when polymerization rate is increased, favoured by high monomer concentration and irradiation dose, the flux declined. This decline was potentially caused by polymerization occurring into PA free-volume. At 10 and 35 v/v% of monomer concentration, the water flux tended to severely decline with increasing irradiation dose. The mass gain was found to increase more rapidly from 0.6 to 3.5 wt% at 10 v/v%, while at 35 v/v%, the mass gain was increased from 2.8 to 14.4 wt% between 1 and 100 kGy irradiation total doses respectively. The flux was shown to be dependent on the resultant grafted coverage. In another work, using conventional chemical route, showed a similar impact of excessive coating layer on PA TFC membranes. The increasing monomer concentrations led to increased grafted film with a thickness estimated at ~300 nm which in turn caused flux decline of 81%^15^. As expected, this trend demonstrated that polymerization kinetic rates were favored with increasing monomer concentration and total irradiation dose. As the membrane resistance to water increased with increasing monomer concentration and irradiation total dose, the salt rejections were also influenced. The salt rejection capability of the membranes was assessed systematically to evaluate the impact of both the solution compositions and the total irradiation dose on the materials integrity.

At low VIM concentrations, 1 v/v%, the salt rejection of the membranes was found to be similar to that of the pristine membranes for all irradiation doses and of the order of 96% (Fig. 1d). The salt rejection across the grafted membrane with 1 v/v% of VIM concentration fluctuated between 96.7% and 95.7% between 1 and 100 kGy, compared to 98% obtained for the pristine membrane. Salt rejection values were however found to start declining upon exposure to higher VIM concentrations. At a concentration of 10 v/v% VIM, the salt rejection declined from 95% to 92.5% and 94.5% at 1, 10 and 100 kGy, whereas at a concentration of 35 v/v% of VIM, salt rejection strongly declined from 92% to 84% between 1 and 100 kGy total dose respectively. These salt rejection results show that grafting at a concentration of 10 v/v% or more leads to substantial loss in the membrane’s ability to reject salt. Salt rejection is governed by both surface charge across the surface and free volume distribution within the material^2^. The causes for the salt rejection decline may be attributed to several factors including free volume alterations within the PA material^9^.

However, in this study the water flux alteration associated with mass gain is here presented as an empirical demonstration of free-volume alteration promoted by the surface grafting. Such variations may be directly associated with VIM molecules and empowered upon irradiation. To assess the impact of the potential sources of degradation, permeation tests were performed on the series of pristine samples. The samples in the MeOH/water mixture (Figure S1a), without VIM monomers, were exposed to the different irradiation total doses. These samples demonstrated that irradiation alone did not damage the PA layer regardless of the irradiation doses with minimal changes in salt rejection. A previous study on gamma-ray irradiation of RO membranes immersed in saline solution showed that flux and salt rejection was practically maintained when membranes were irradiated at 100 kGy due to PA resistance to irradiation and integrity of the amide groups^16^. On the other hand, the performance of the membranes exposed to the monomer solutions alone (Figure S1b) led to significant permeation increases of ~50% at low VIM concentrations, between concentrations of 1 and 10 v/v%, but at a concentration of 35 v/v% of VIM the flux was increased by only 19%, compared to pristine membrane (45.9 L.m^−2^.h^−1^). This effect is potentially caused by the swelling of the PA layer upon monomer uptake within the materials free volume. The NaCl rejection across the membranes however progressively declined as a function of increasing monomer concentration from 97 to 92% at concentrations of 1 and 35 v/v% of VIM respectively. This trend indicates that the PA layer was significantly affected by exposure to the VIM monomer molecules over 10 v/v% in concentration. The mass gain confirms that for this series the monomer molecules were adsorbed and mass values were found to increase with increasing monomer concentration from 0.4 wt%, for both 1 and 10 v/v% VIM concentrations, to 2.7 wt% for 35 v/v%. These results indicate that the monomer solutions potentially diffused across the material towards the poly(sulfone) (Psf) layer upon longer exposure durations leading to higher mass gains even after samples being washed before this analysis.

### Morphology analysis of grafted layer and PA surface integrity

**Figure 2:**
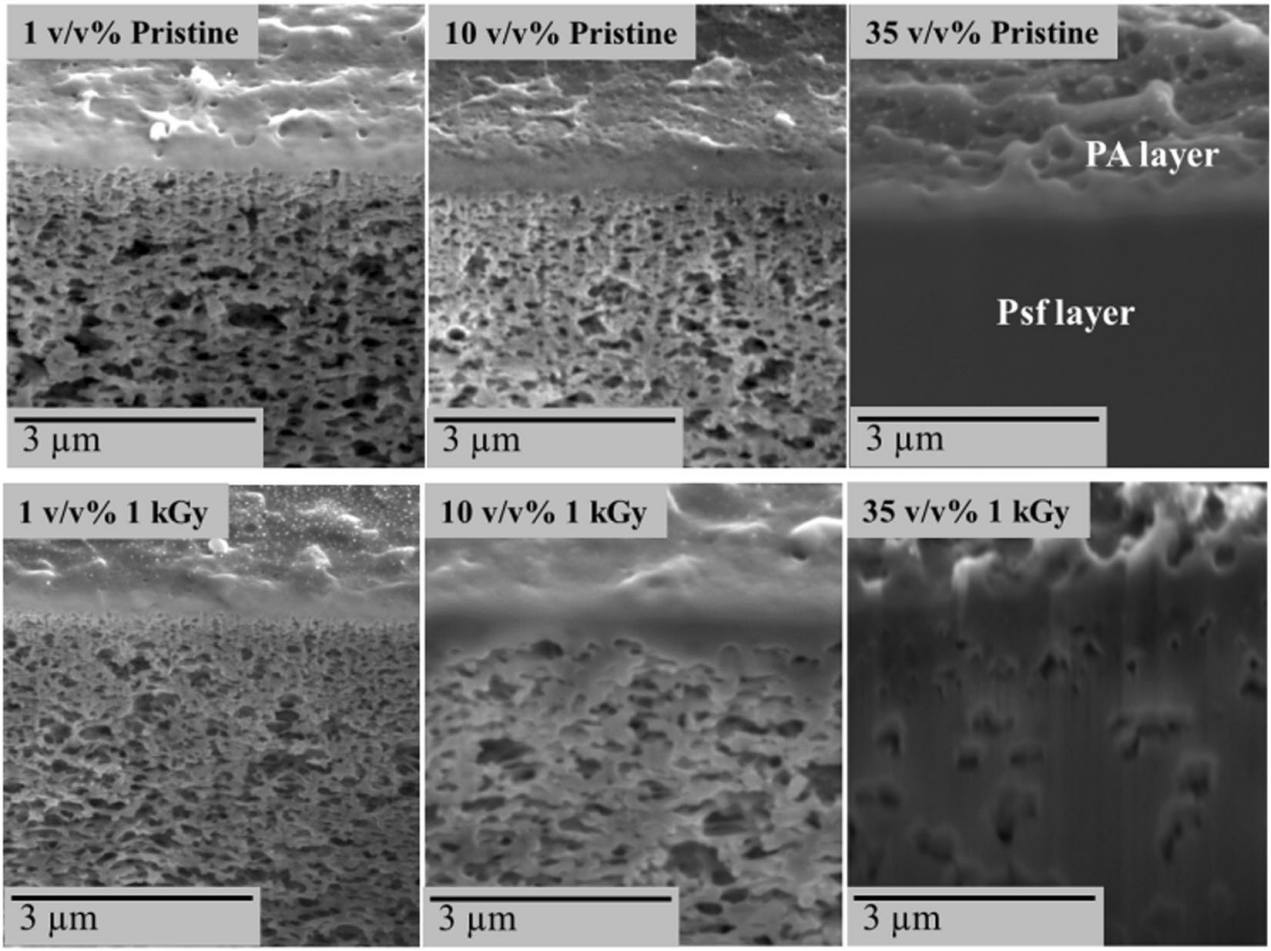
Cross sections of TFC membranes at a fixed dose of 1 kGy. Top: series of pristine samples exposed to VIM solution and no irradiation and bottom: corresponding irradiated membranes.

The morphologies of the grafted functional layers formed by irradiation polymerization were analyzed by their SEM cross sections (Fig. 2). Cross sections of the samples were prepared to evaluate the extent of grafting of the monomer across the PA and Psf layers. At 1 kGy irradiation dose the PA layer was found to be denser and thicker with increased monomer concentration compared to the pristine membranes.

When irradiation is increased to 100 kGy (Figure S3) the densification is also intensified specially with a 10 v/v% of VIM concentration compared to the pristine membrane. Excessive polymerization was also found to occur across the pores of the Psf layer and appeared to progressively obstruct the pores at higher concentrations. Furthermore, SEM images and (Figures S2a and S4) and AFM (Figures S2b and S5) were used to evaluate the resultant morphological changes across the surface of the PA layer. The morphology of the pristine membrane materials was found to be irregular and rough as previously reported (Figure S2a)^17^.

However, increasing the monomer concentrations and the irradiation total doses tended to smoothen and flatten out the natural protrusions present across the native PA surface (Figure S4). At low irradiation doses, between 1 and 10 kGy, although different levels of surface texturation were observed, protrusions were significantly reduced at 35 v/v% of monomer concentration suggesting an intensified polymerization process between the surface protrusions. In previous work, similar morphology alterations were reported when TFC membranes were exposed to other irradiation techniques such as low pressure plasma surface modification. The performance of the materials were however, strongly reduced, which was attributed to the collapse of the supporting layer pores upon evaporation of solvent during the low pressure plasma operation. The membranes, exposed to the monomer molecules or simply to the reactant gas environment, were found to be progressively etched with increasing plasma density leading to lower separation performance^7, 13, 18^. Therefore, the route proposed with gamma-ray irradiation, operated at 1 atm and in mild conditions, offers a clear advantage compared to these previous works.

Across the series of pristine membranes exposed to monomer solutions without irradiation, these protrusions appeared largely unaffected and only visually slightly flattened which could be due to surface tension effects caused by the MeOH/water in solution upon drying and evaporation. Another series of pristine samples exposed and irradiated with only MeOH/water, the solvent system used to prepare the VIM solutions (Figure S4), showed that this solvent system tended to slightly flatten these protrusions generating a smoother layer, with however, no significant variations between the series of irradiated samples. In this respect, the PA material was shown to be vulnerable to oxidative reagents exposure and therefore to potential degradation and chemical etching, which may have contributed to the flattening of the surfaces.

The levels of texturation were calculated from AFM maps by measuring the average roughness (Ra) for the samples, as presented in Figure S5. The surfaces of the grafted membranes were smoothened and roughness values found to decrease with increasing irradiation dose. At 1 kGy, the roughness was reduced compared to the pristine membranes by over 50% and found to plateau around 36 nm for the samples grafted with VIM concentration solutions at 1 and 10 v/v% respectively. The roughness of the materials was slightly higher, at 45 nm, for the 35 v/v% irradiated VIM concentration solution, yet approximately 30% lower than the pristine membranes, suggesting excess build-up polymerization due to the prolonged irradiation conditions. At 10 kGy, a similar plateau was found between 1 and 10 v/v% with roughness around 44 and 42 nm respectively. However, at 35 v/v% concentration, the roughness of the samples was significantly decreased to 24 nm. At 100 kGy, increasing monomer concentration to 1, 10 and 35 v/v% led to lower roughness values measured at 34, 35 and 27 nm respectively.

The calculated roughness values of the series of pristine membranes exposed to monomer solutions without irradiation demonstrate that sole contact with the VIM monomer smoothen the membranes surface. The roughness of the membranes exposed to 1, 10 and 35 v/v% of VIM concentration was decreased from 60.5 nm (pristine membrane Figure S2b) down to 27, 37 and 32 nm respectively. Monomer molecule adsorption was found to affect the mass gain and is in agreement with the measured reduced roughness across the series of pristine membranes exposed to monomer solutions alone. Therefore, potential chemical etching was also confirmed with VIM pristine series without being irradiated. However, further chemical analysis is necessary to confirm grafted layer and to analyze the impact of irradiation on the nascent functional groups of the membranes.

### Chemical analysis of grafted layer and PA surface integrity

The distribution of the amine groups across the grafted surfaces was assessed by XPS analysis. The survey analysis detected that elemental nitrogen at% was progressively increased with increasing VIM concentration. However, this increase was also detected in pristine series exposed to the monomer solution solely, which also confirms the impact of monomer adsorption as detected with mass gain and morphology analysis. The N/C ratio therefore was increased from 0.03 for the pristine membrane up to 0.26 for the membranes irradiated at 35 v/v% and 100 kGy of total irradiation dose (Tables S1 to S4). N1S peak were deconvoluted to identify the different functional groups generated upon grafting and the nature of the reaction pathways leading to the amine enrichment (Figure S6). In terms of PA integrity, grafting of amine functionalities caused peaks dissociations within the C1s main peak, which are attributed to changes across the amide bonds present across the nascent PA. (Figure S6). The peak at 285.98 eV corresponding to C-N sites, across the PA, was significantly decreased after grafting, indicating consequent damage or reconfiguration of the PA structure^16, 19^. Peaks appeared at around 291 eV, corresponding to π-π* - shake-up transitions of aromatic structures. Such peaks were found only for the grafted samples at 10 and 35 v/v% VIM concentrations. The presence of these peaks is an indication of potential remaining monomer aromatic structures adsorbed across the surface^20^. The π-π* - shake-up transitions was therefore used as a reference to detect residual imidazole rings, which should have been opened, due to low dissociation energies, upon polymerization^21^.

The XPS results were confirmed by FTIR spectra analysis (Figure S6), demonstrating that new chemical bands, likely belonging to the amine groups, appeared upon irradiation of the membranes in the VIM solutions. FTIR is an extremely sensitive technique able to probe >100 nm within materials, and is therefore able in the present study to probe over the whole thickness of the PA layer within the TFC membrane^22^. The absorption of the band at 3330 cm^−1^ corresponding to N-H and/or O-H groups stretching vibrations in the neighboring of the PA aromatic rings^23^, significantly increased after grafting suggesting an increase of the density of amine groups across the surface of the membranes. This broad absorption region at 3330 cm^−1^, which is particularly enhanced after grafting at 35 v/v% of VIM concentration and upon irradiation at 100 kGy, is here attributed to the presence of hydroxyl groups resulting from the presence of remaining MeOH or water solvent molecules. Azole groups particularly, as present across the VIM monomer molecules, were previously reported to have a very strong affinity to adsorb water molecules and therefore led to the broad band effect^24^. The presence of π-π* - shake-up transitions peaks, as discussed in the XPS section, also indicated residual unreacted VIM aromatic structures caused by an excess of monomer molecules. Therefore, the broad 3300 cm^−1^ band (Figure S7) is expected to be an indication of residual aromatic structures from the VIM at high VIM concentration. An extra band at 3110 cm^−1^ appeared upon increased monomer concentration and irradiation dose which was attributed to stretching vibrations of C=CH and N=CH bonds from the imidazole ring^24^. In this regard, the imidazole groups were partially dissociated. The absorbance of the bands corresponding to functional groups in the vicinity of the aromatic amide bands at 1663, 1609 and 1545 cm^−1^ were also enhanced after irradiation grafting suggesting attachments of amine groups from the grafting layer (Figure S8). However, such bands in 35 v/v% at 100 kGy shows an outlined spectra suggesting chemical degradation which explains significant declined salt rejection. Furthermore the appearance of a band at 1649 cm^−1^ found to increase in intensity with monomer concentration, which is here likely correlated to a direct and measurable increase of the primary amine pendant groups surface density^25^. This increase was found to be more prominent after exposure at 35 v/v% of VIM concentration on both irradiated and pristine membranes solely exposed to the monomer molecules. This finding therefore demonstrates that in addition to being covalently grafted to the surface, monomer molecules were also physically adsorbed and up-taken within the PA material, corroborating the mass gain findings. The quantity of monomer present within the free volume of the PA material is however not possible to be evaluated. The changes visible across the pristine series however indicate that the quantity of free molecules is rather limited. In addition, the enhanced intensity of the band at 918 cm^−1^ (Figure S9), attributed to C-H out-of–plane bending, is attributed to dissociated vinyl groups from the imidazole ring, which was dissociated upon irradiation^26^. The irradiated MeOH/water pristine membranes did not show chemical changes in these specific bands which therefore suggests, as expected, that no polymerization occurred without gamma ray irradiation, and that up-take of the monomer was likely a purely physical-sorption phenomenon.

### Analysis of surface charge and impact on membrane performance

The chemical nature of the grafted surfaces also influenced the resultant net surface charge. In the present study, the grafted amine functional groups dramatically altered the surface polarity, shifting the net charge from highly negative to largely positive. Streaming potential assessments were performed for the 1 v/v% concentration series for increasing irradiation doses as a model since these membranes exhibited the most enhanced performance compared to the reference materials^18^.

**Figure 3:**
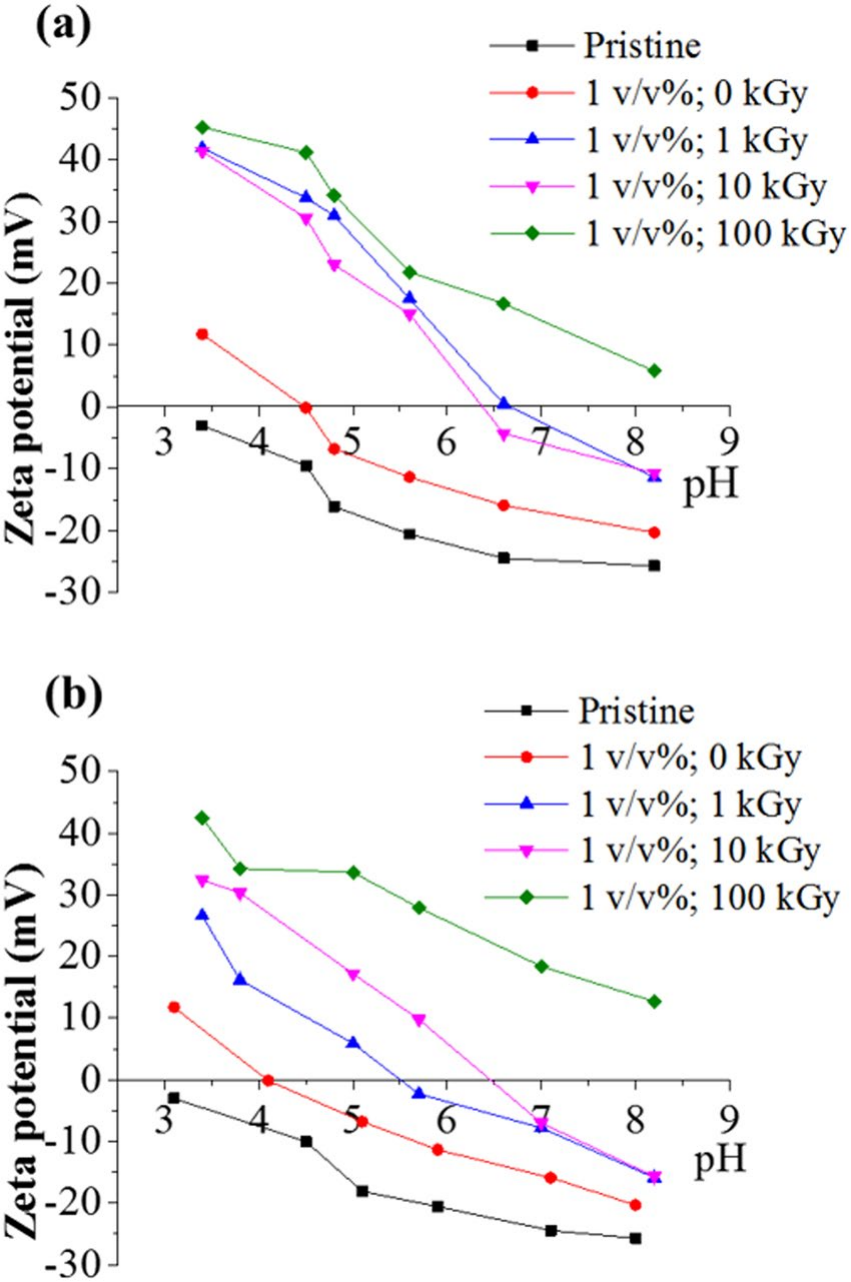
Surface charge of amine grafted membranes using 1 v/v% of the VIM monomer (**a**) before permeation tests and (**b**) after permeation tests.

Electrostatic interactions between the solution and the surface ionizable functional groups (R-COO^−^, R-NH^3+^) result in protonation and deprotonation of amine and carboxylic groups respectively as a function of the pKa of the moieties, and therefore as a function of the test solution’s pH. The grafted membranes presented in Fig. 3a were shown to present an increasing density of surface amine functional groups which led to a net increase of their surface positive charge. As a reference – as supplied membranes typically exhibit a negative charge profile from almost zero to −25 mV across pH range of 3 to 8 respectively^18^. The grafted membranes presented highly positive net charge profiles across the full pH range. The isoelectric point (IEP) of the grafted membranes between the 1 and 10 kGy was shifted from approx. pH 4.5 to pH 6.8 reaching a net charge of +45 mV in the acidic pH range. In addition, the net charge profile in the alkaline pH range above pH 8, was found to reach −5 mV, therefore nearly 5 times higher than the reference membranes. At 100 kGy, the charge was always positive across the complete pH range, reached maximum of +47 mV in the acid range and a minimum of +5 mV within the alkaline range. These results are significant since they provide evidence that TFC materials may be in-depth modified to offer highly tunable surface charges and IEPs. These are, to the best of the authors’ knowledge, the largest net charge shifts ever reported for TFC PA based membranes. On the other hand, the pristine membranes exposed to a 1 v/v% of VIM concentration solution, have up-taken monomer molecules by adsorption, presented an IEP at pH 4.5 and a positive net charge increased range from pH 3 to 6. The net difference with the reference, untreated, membranes was therefore largely significant in that range. However, within the alkaline range, and particularly above pH 8, the pristine membrane exhibited a negative profile with a net charge around −15 mV, within the same range to that of the pristine membranes, demonstrating more similarities with the reference membrane.

Furthermore, in order to further evaluate the stability of the grafted molecules, the samples were also tested after permeation testing to assess for potential delamination or desorption of functionalities which may occur within the module test due to the high shear rate (2.4 m. s^−1^) and the high pressure exerted across the thickness and top layer of the membranes. Indeed in pressure driven separation, the resistance of the membrane is largely concentrated upon the PA layer. After permeation (Fig. 3b), charge profiles of all grafted membranes started to return to the pristine membrane charge profile. Their IEP points shifted towards acidic range by approximately ± 1.0 pH unit, while the reference membranes exposed only to a to 1 v/v% of VIM concentration shifted by ± 0.5 pH unit. Although the positive charges were reduced for amine grafted membranes they still presented a high content of amines with charges reaching between +30 and +45 mV. The high positive charge TFC membranes offer practical value when treating liquids containing positively charged molecules such as proteins, surfactants or heavy metal ions. The control of the IEP position has a significant effect on selectivity, because it dictates the charge on the functional groups of the membrane material and of the molecules in solution^27^.

The charge of the material may also offer low-adherence with contaminants and superior recovery capabilities as previously demonstrated^28^. Resultant chemical alterations affecting wettability were also accessed by water contact angle measurements. The contact angle is a sensitive tool that is strongly affected by the surface charge and topology. The contact angle is correlated with the water molecules orientation at the solid-liquid interface.

**Figure 4:**
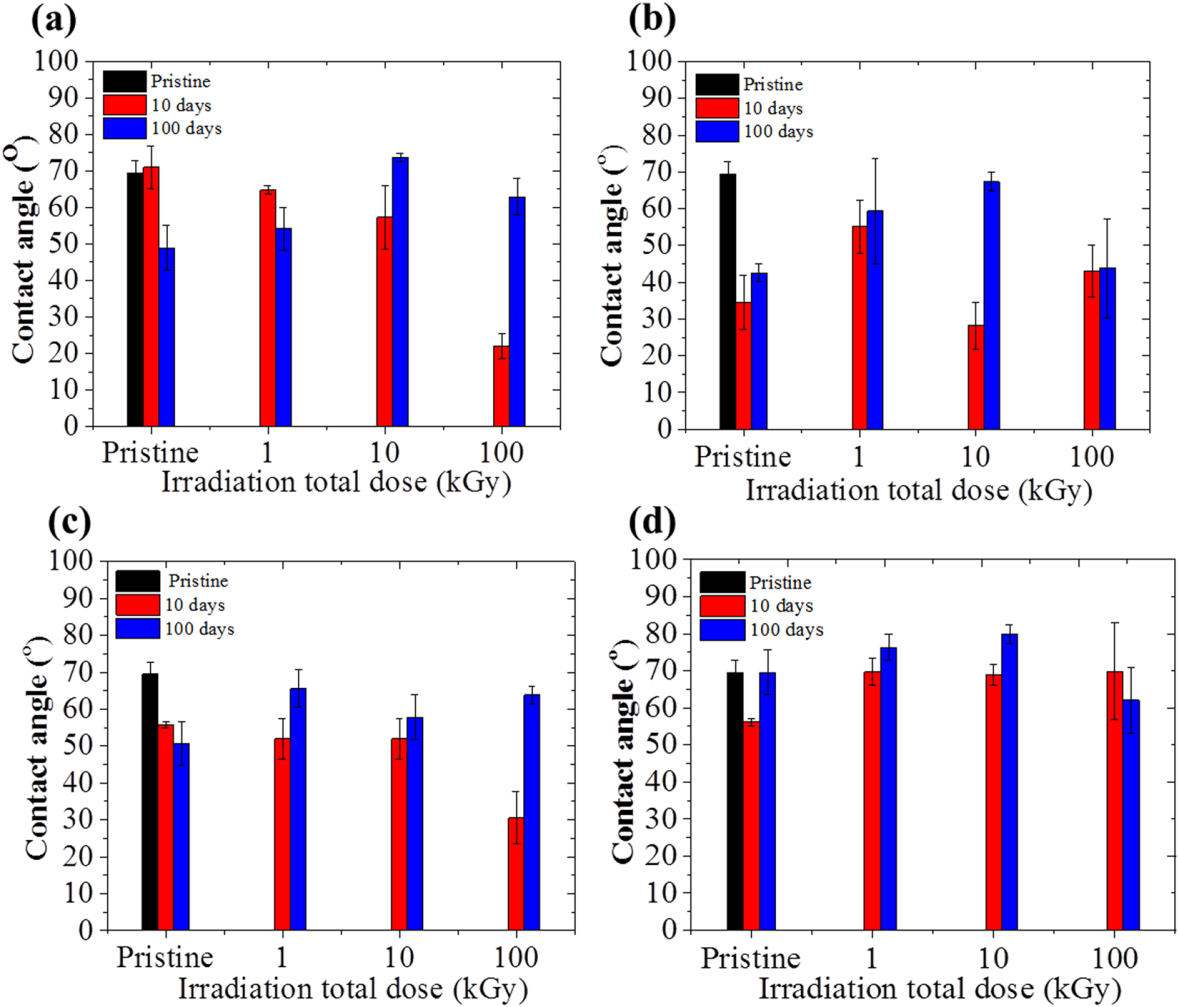
Water contact angle of membranes exposed to (**a**) 1 v/v% VIM solution, (**b**) 10% VIM solution, (**c**) 35% VIM solution and (**d**) MeOH/water.

For instance, a positively charged surface with a high amine functional groups content would exhibit a higher fraction of ionized species across a low pH ranging from pH 2 to pH 6^29^. The protonated species on the surface of the materials would therefore enhance the net positive charge and promote Coulomb interactions thus attracting the negatively charged dipoles, such as water^29^. Such interactions may increase hydrogen bindings between the surface and water and in turn, reduce the dynamic wettability of the material^29^. Considering that the contact angle was measured at pH 6 and at this pH the surface is positively charged, the contact angle after grafting tended to be decreased (Fig. 4). The contact angle of the grafted membranes at 1 v/v% of VIM concentration (Fig. 4a) showed consistent decrease with increasing irradiation dose. The membrane exposed to 1 v/v% VIM concentration but to no irradiation showed similar behaviors to that of the pristine membranes, measured at 70 ± 5.90°, while after grafting it was shown to progressively decrease to 64.7 ± 1.2o° and 2 2 ± 3.5o° from 1 to 100 kGy, respectively. These results correlate well with the surface charge trend previously discussed where the positive charges were also increased with increasing irradiation dose.

Therefore, increasing irradiation dose promoted more amine contents. However, the formation of aliphatic amines due to imidazole rings opening promoted by the high irradiation total dose cannot be discarded. Indeed, FTIR analysis detected bands at 1649 cm^−1^ and 918 cm^−1^ suggesting the presence of primary amines and C-H groups (Figures S8 and S9). Aliphatic amines were shown to lead to higher states of ionization than in aromatic form and therefore lead to an increase of the discussed effect^29^. On the other hand, Fig. 4d shows that the samples from the series of pristine membranes irradiated in MeOH/water solution without monomers were not significantly altered, and presented contact angles consistent around 70° across the series of irradiation total doses. This confirms the results presented in FTIR, XPS and performance test where these pristine membranes showed no significant changes even at high doses of irradiation (Figure S1a). The stability of the water contact angle was also assessed by measuring the contact angle on the same samples with an interval of 100 days. Interestingly, a slight tendency for hydrophobicity recovery was found to occur, especially for the samples grafted at higher irradiation doses. This result, in line with previously discussed surface charge IEP shifts, suggests that a portion of the amine functional groups may have suffered molecular re-orientation.

In order to confirm the impact of the surface charge on the membrane performance, tests were performed with divalent cations, Ca^2+^, based solutions. The salt rejections with the CaCl_2_ solutions were found at 99%, significantly higher than those reported for the NaCl solutions. The grafting series irradiated at 1 kGy with increasing monomer concentration was chosen to demonstrate the effect of Donnan layer exclusion since this series showed a variety of salt rejection values in NaCl desalination test. The stability of the coating was also evaluated by assessing the surface charge after desalination tests. The surface charge of membranes tested for desalination of CaCl_2_ at 1 kGy total dose and for 1, 10 and 35 v/v% VIM concentrations, was also significantly altered and had the IEP shifted towards 6.8 and overall net surface charge reaching up to +45 mV. The respective pristine series exposed to 1, 10 and 35 v/v% showed the effect of monomer adsorbing where IEP point was found at 4.5 for 1 and 10 v/v% membrane and 3.5 for 35 v/v% membrane. The net surface charged reached a maximum of +20 mV with 10 v/v% monomer concentration (Figure S10). Although monomer adsorption intrinsically affected the surface charge, the irradiation grafting significantly contributed to the charge increase and durability.

As previously discussed, NaCl rejection at 1 kGy of 96%, 95% and 92% and fluxes of 70, 68 and 55 L.m^−2^.h^−1^ were obtained for the 1, 10 and 35% v/v% VIM concentration solutions respectively. The salt rejection in RO membranes is primary driven by Donnan layer exclusion than salt diffusion^30^. The same tested series of 1 kGy with increasing VIM concentrations showed an increase of CaCl_2_ rejection as high as 99% for flux values around 46.7 L.m^−2^.h^−1^. Considering that the free volume of commercial TFC PA membranes are typically reported in the range of ~0.259 to 0.289 nm hole-radius^2^, the high selectivity presented indicates that strong electrostatic interactions occurred upon separation once the ionic radius of Ca^2+^ and also Na^+^ ions is 0.99 Ǻ with diameter ~0.2 nm^31^. This high salt rejection therefore, demonstrated the integrity and stability of the coatings across the materials as well as the potential of the novel technique to alter commercial membranes towards high permeability and high selectivity materials.

## Conclusions

Irradiation-induced grafting was systematically investigated and the altered surface properties were critically correlated to TFC membrane performance. The nanoscale PA network was shown to be a limiting factor to control the degree of grafting. Considering the structural characteristic of PA ultrathin layer, grafting with low VIM concentration of 1 v/v% at 1 kGy irradiation dose was demonstrated to be sufficient to alter the membrane from negatively to positively charged. The selectivity with NaCl was maintained around 96.7% while the rejection of CaCl2 was dramatically improved to >99% which was favored by improved electrostatic interactions. On the other hand, flux was likely to be compromised upon densification of PA layer and grafting depth at the Psf layer upon longer treatment exposure durations. Such effects tended to increase membrane transport resistance to water and were intensified at high doses and VIM concentrations. This work therefore offers for the first time a simple and highly up-scalable route to tuning the selectivity of TFC RO membranes by homogeneously altering the overall membrane surface charge, opening the route to custom-designed TFC membrane materials suited to specific industrial applications.

## Electronic supplementary material

Supplementary Information
