# Supplementary material for: Charge tunable thin-film composite membranes by gamma-ray triggered surface polymerization
Source: Sci Rep. 2017 Jun 30;7:4426. doi: 10.1038/s41598-017-04900-5 (PMC5493691; doi:10.1038/s41598-017-04900-5)
Supplement: Supplementary file 1 — Supplementary Information [file 41598_2017_4900_MOESM1_ESM.doc]

Charge tunable thin-film composite membranes by gamma-ray triggered surface polymerization

Rackel Reis^†‡*^, Mikel C. Duke^†^, Blaise L. Tardy^§^, Daniel Oldfield^#^, Raymond R. Dagastine^§^, John D. Orbell^†^, and Ludovic F. Dumée^‡*^

†Institute for Sustainability for Innovation, College of Engineering and Science, Victoria University, Melbourne, VIC 3030, Australia

‡ Deakin University, Institute for Frontier Materials, Geelong, VIC 3216, Australia

# RMIT, School of Science, Applied Science, Melbourne, VIC 3000, Australia

§ Department of Biomolecular and Chemical Engineering, The University of Melbourne, VIC 3010, Australia

# School of Applied Sciences, RMIT University, Melbourne, VIC 3030, Australia

[*Corresponding authors: rackel.reis@live.vu.au.edu](mailto:*Corresponding authors: rackel.reis@live.vu.au.edu); [ludovic.dumee@deakin.edu.au](mailto:ludovic.dumee@deakin.edu.au)

**Supplementary Materials**

Radiation grafting was performed at the Gamma Technology Research Irradiator (GATRI) Facility at the Australian Nuclear Science and Technology Organization (ANSTO). The radiation source was cobalt-60 (^60^Co) offering a dose rate of 2.35 kGy/h, determined by reference dosimetry analysis in a previously reported procedure^8^. The dosimeters used during the grafting process were Red Perspex/4034NC and Amber Perspex/3042Y. The membranes were placed in PE bags, with a tolerance level of 1,000 kGy^9^ and filled with the grafting solutions, as described below. The plastic bags were sandwiched between two glass plates to ensure homogeneous wetting of the membrane materials during irradiation, and packed in a box with the dosimeters. The experiments were performed at an ambient temperature of ~24 ^o^C. The overall uncertainty associated with an individual dosimeter reading was calculated by the ANSTO technicians to be 4% providing a level of confidence of approximately 95%. The uncertainty evaluation was carried out in accordance with the ISO guide for the expression of uncertainty in measurement.

The grafting process was performed for total irradiation doses of 1, 10 and 100 kGy. Giving that the dose rate was equivalent to 2.35 kGy/h, the irradiation total doses of 1, 10 and 100 KGy corresponded to 0.4, 4 and 42 h of irradiation, respectively. The irradiation-induced grafting was performed with pre-conditioned membranes without drying step, immersed in three different VIM solutions dissolved in MeOH/water at 50 v/v %. A series of control membranes were prepared using wetted control membranes (stored in SMBS as described in Section 3.2.1). Pristine membranes exposed to VIM solutions without irradiation in order to simulate the direct impact of the monomer for a duration equivalent to that of the highest irradiation total dose of 100 kGy of 42 h. The series of MeOH/water solution assessed the direct impact of the radiation on the membrane surface.

The mass gain in this study was used as a quantitative tool to accessing polymerization which occurred across the surface of the treated material^10^. After irradiation, the membranes were thoroughly washed with deionized water, in order to remove any remaining non-polymerized materials, and dried for 1 h at 65oC followed by weighing on a Metler MS40025/01 balance (+/- 0.001 mg). The irradiated samples were cut into 7 X 6 cm pieces to provide a manageable surface area of 42 cm^2^ (+/- 0.3 cm^2^).

The degree of grafting was calculated using Equation 1:

% Grafting =
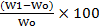
 (1)

Where W_o_ and W_1_ are the dry weights of the control membrane and the grafted membrane respectively.

The membranes exposed to the monomer solutions simulated the same duration as 100 kGy irradiation dose which therefore corresponded to 42 h of exposure. On the other hand, membrane exposed to MeOH/water, but not irradiated, exhibited similar performance as pristine membrane within 1.5% of variation tolerated for such materials. A slight drop of flux of 13% from 45.9 L.m^-2^.h^-1^ to 40 L.m^-2^.h^-1^ was found between after 42 h of contact with MeOH/water. In terms of salt rejection, this membrane series showed that selectivity was always maintained around 97 – 97.5% range of the pristine membrane and this at all irradiation doses.


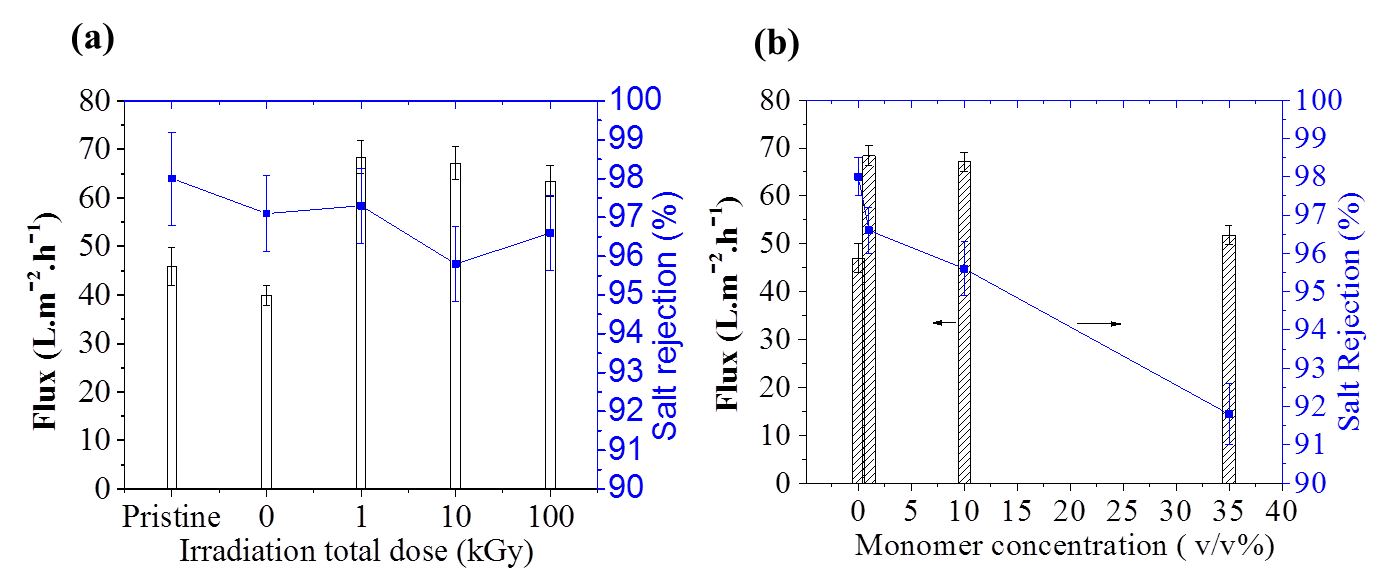


Figure S1: Series of reference membranes a) membranes exposed to MeOH/water solution and increasing irradiation doses and b) membranes exposed to monomer solutions (1, 10 and 35 v/v%).

SEM and AFM analysis evaluated the resultant morphological changes at the PA layer. The morphology of the nascent membrane materials was found to be irregular and rough as previously reported (Figure S2a S2b) [12].


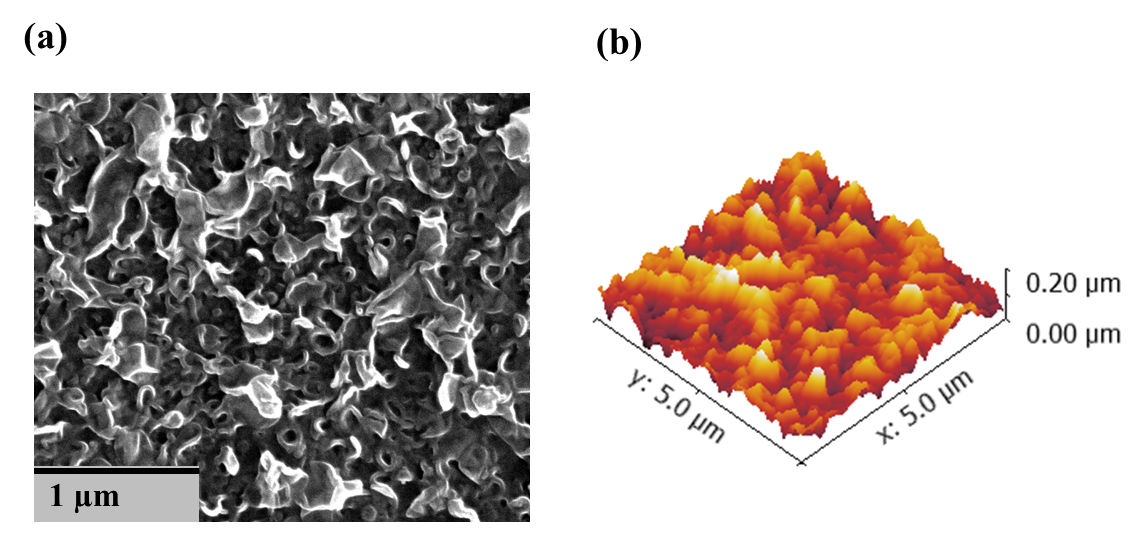


**Figure S2:** a) SEM image of pristine membrane and b) Surface roughness map of pristine membrane measured by AFM analysis.

When irradiation is increased to 100 kGy (Figure S3) the densification is also intensified specially with a 10 v/v% of VIM concentration compared to the pristine membrane. Excessive polymerization was also found to occur across the pores of the Psf layer and appeared to progressively obstruct the pores highlighting the kinetic dependence of the polymerization process.

***
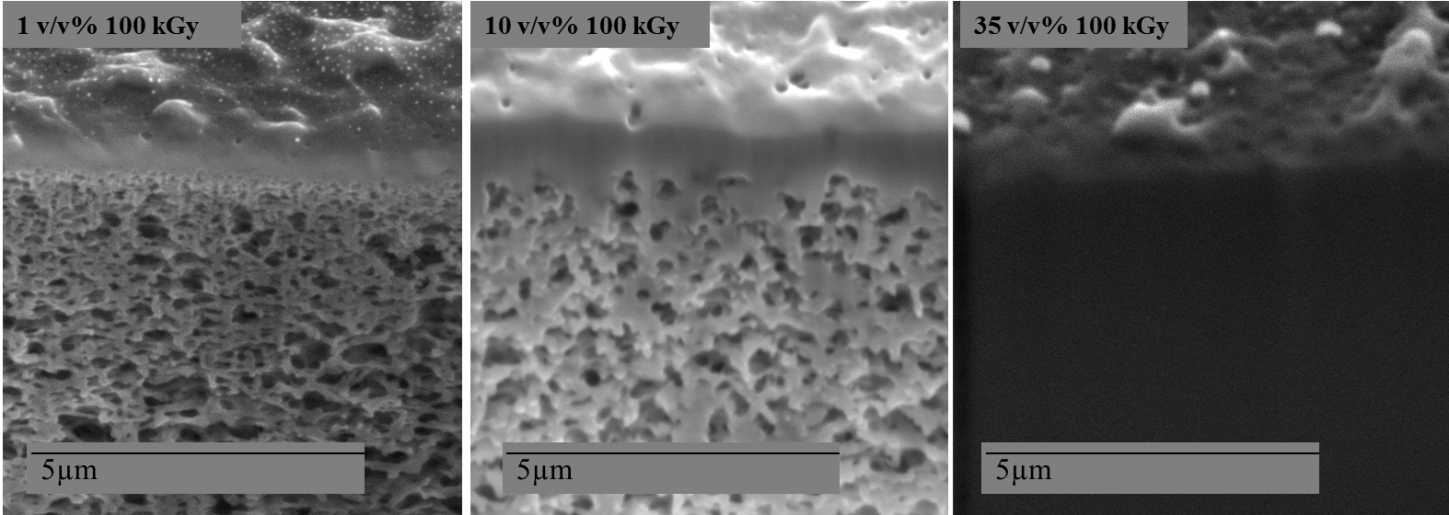
***

**Figure S3:** Cross sections of TFC membranes at a fixed dose of 100 kGy at different VIM concentration doses

The morphology of the samples was strongly altered with the irradiation polymerization. Increased monomer concentrations and irradiation total doses tended to flatten the natural protrusions present across the native PA surface.


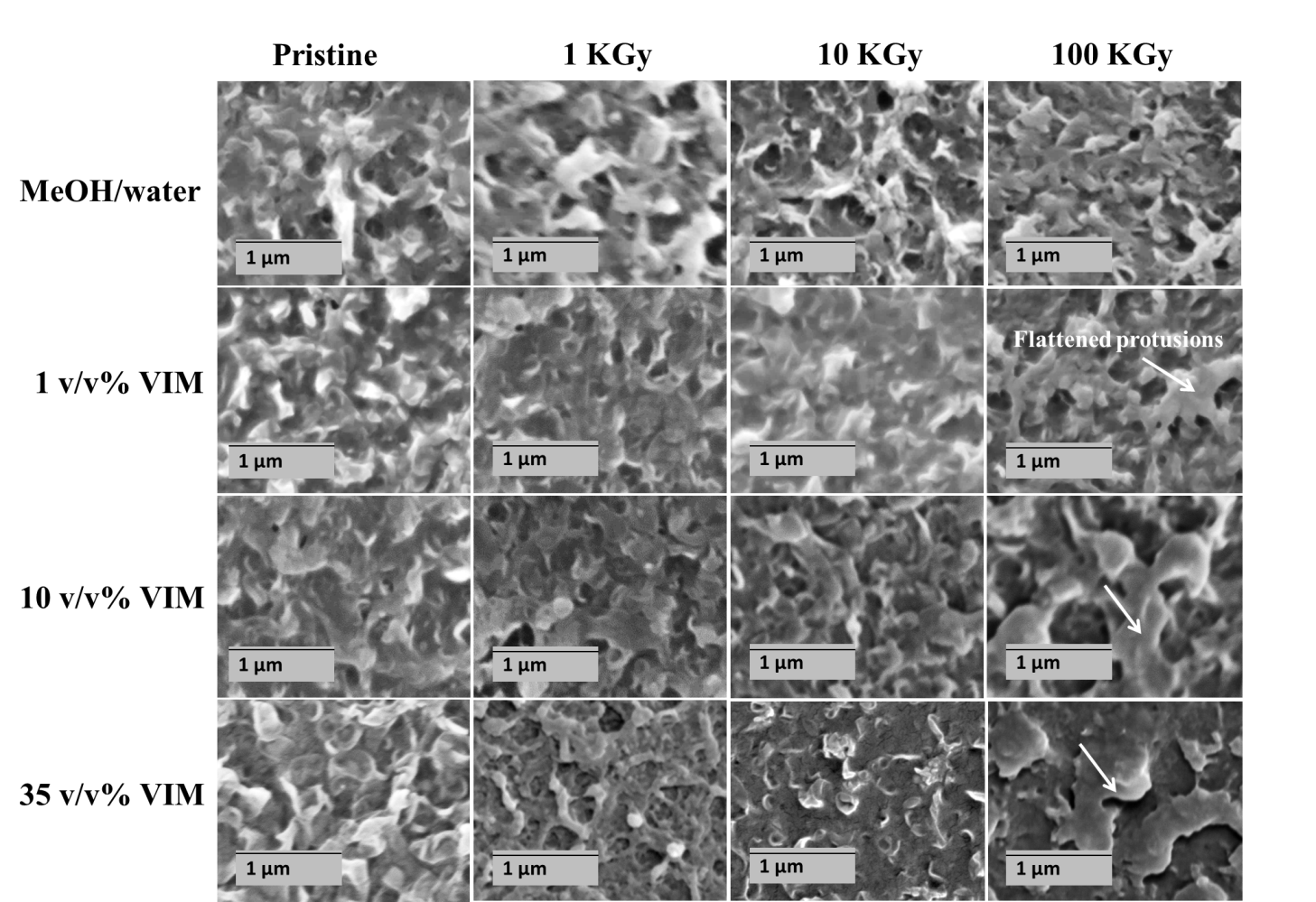


Figure S4: Surface morphology changes observed by SEM with 1 µm scan size.

The levels of texturation were calculated from AFM maps by measuring the average roughness (Ra) for the samples, as presented in Figure S2. The calculated roughness values of the series of control membranes exposed to monomer solutions without irradiation demonstrate that sole contact with the VIM monomer smoothen the membranes surface.

***
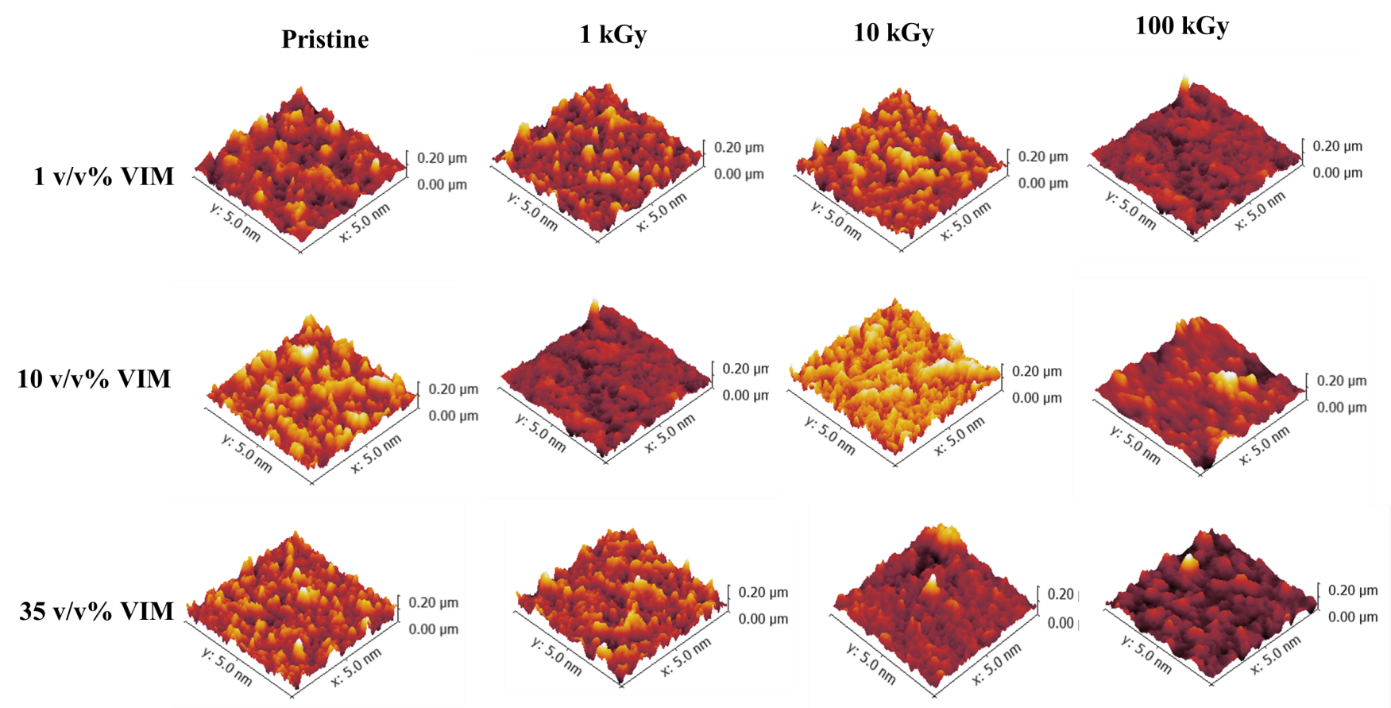
***

Figure S5: Surface roughness measured by AFM with 5 x 5 µm scan size

The survey analysis detected that elemental nitrogen at% was progressively increased with increasing VIM concentration with N/C ratio increased from 0.03 for the pristine membrane [14] up to 0.26 for the membranes irradiated at 35 v/v% and 100 kGy of total irradiation dose (Table S1 to S4).

***Table S1:*** XPS elemental analysis of control membranes

|  | **Sample** | **Cat%** | **O at%** | **N at%** | **N/C** | **O/C** |
| --- | --- | --- | --- | --- | --- | --- |
| **O kGy** | **MeOH/water** | 82.1 | 14.3 | 3.7 | 0.04 | 0.17 |
|  | **1 v/v%-VIM** | 74.7 | 20.4 | 4.9 | 0.06 | 0.27 |
|  | **10 v/v%-VIM** | 75.0 | 17.8 | 7.2 | 0.09 | 0.23 |
|  | **35 v/v%-VIM** | 76.4 | 17.6 | 5.9 | 0.08 | 0.23 |

***Table S2:*** XPS elemental analysis of grafted membranes

|  | **Sample** | **Cat%** | **O at%** | **N at%** | **N/C** | **O/C** |
| --- | --- | --- | --- | --- | --- | --- |
| **1 kGy** | **MeOH/water** | 79.9 | 14.5 | 5.6 | 0.07 | 0.18 |
|  | **1 v/v%-VIM** | 73.6 | 17.8 | 8.6 | 0.12 | 0.24 |
|  | **10 v/v%-VIM** | 80.7 | 13.7 | 5.6 | 0.07 | 0.17 |
|  | **35 v/v%-VIM** | 72.9 | 20.4 | 6.8 | 0.09 | 0.28 |

***Table S3:*** XPS elemental analysis of grafted membranes

|  | **Sample** | **Cat%** | **O at%** | **N at%** | **N/C** | **O/C** |
| --- | --- | --- | --- | --- | --- | --- |
| **10 kGy** | **MeOH/water** | 78.1 | 18.7 | 2.8 | 0.03 | 0.24 |
|  | **1 v/v%-VIM** | 82.4 | 14.1 | 3.5 | 0.04 | 0.17 |
|  | **10 v/v%-VIM** | 80.7 | 13.7 | 5.4 | 0.07 | 0.17 |
|  | **35 v/v%-VIM** | 77.6 | 14.2 | 5.3 | 0.07 | 0.18 |

***Table S4:*** XPS elemental analysis of grafted membranes

|  | **Sample** | **Cat%** | **O at%** | **N at%** | **N/C** | **O/C** |
| --- | --- | --- | --- | --- | --- | --- |
| **100 kGy** | **MeOH/water** | 75.7 | 21.7 | 2.6 | 0.03 | 0.29 |
|  | **1 v/v%-VIM** | 74.1 | 12.6 | 13.2 | 0.18 | 0.17 |
|  | **10 v/v%-VIM** | 72.1 | 11.4 | 16.6 | 0.23 | 0.16 |
|  | **35 v/v%-VIM** | 74.1 | 6.6 | 19.4 | 0.26 | 0.09 |

On the other hand, grafting of amine functionalities also caused dissociation bonds in the amide region detected by C1s deconvolution. (Figure S5). The peak at 285.98 eV corresponding to C-N sites, across the PA, was significantly decreased after grafting, indicating consequent damage or reconfiguration of the PA structure [14].

***
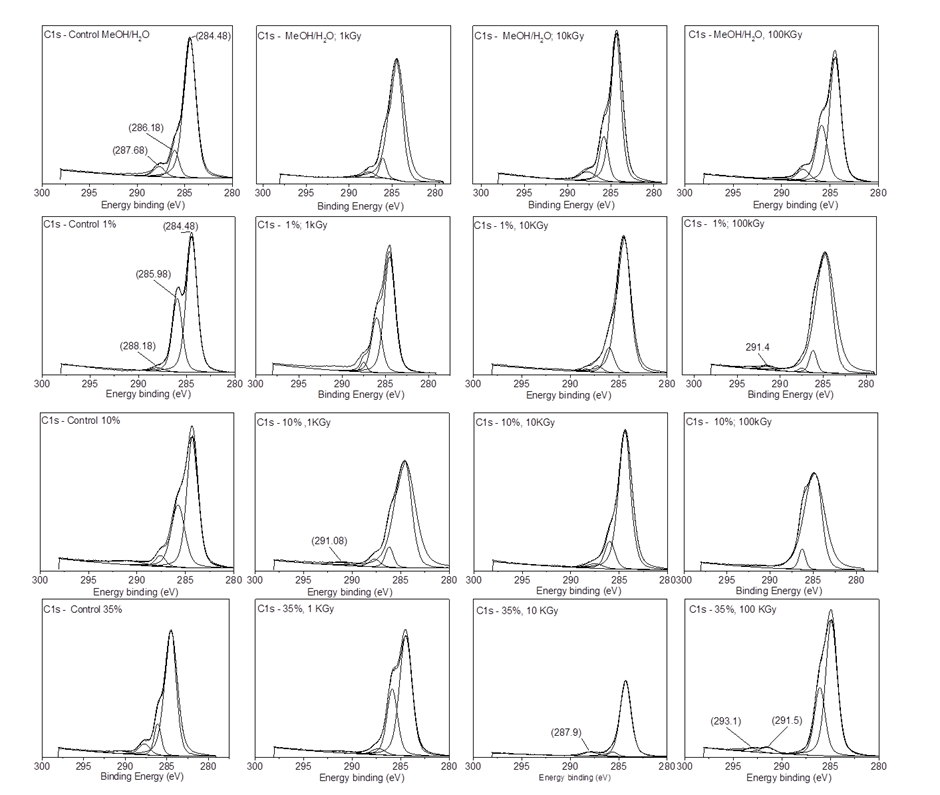
***

**Figure S6:** XPS analysis of C1s deconvolution.

The absorption of the band at 3330 cm^-1^ corresponding to N-H and/or O-H groups stretching vibrations in the neighboring of the PA aromatic rings ^23^, significantly increased after grafting suggesting an increase of the density of amine groups across the surface of the membranes. The broad absorption region at 3330 cm^-1^, which is particularly enhanced after grafting at 35 v/v% of VIM concentration and upon irradiation at 100 kGy, may also be attributed to residual OH groups generated from the MeOH or water solvent molecules. Azole groups, were previously shown to have a very strong affinity to adsorb water molecules and therefore may had led to this band effect [18]. Also as previously discussed in XPS analysis, the presence of π-π* - shake-up transitions peaks may also indicate residual VIM aromatic structures caused by an excess of the monomer in concentration once this band also corresponds to aromatic structures in VIM.


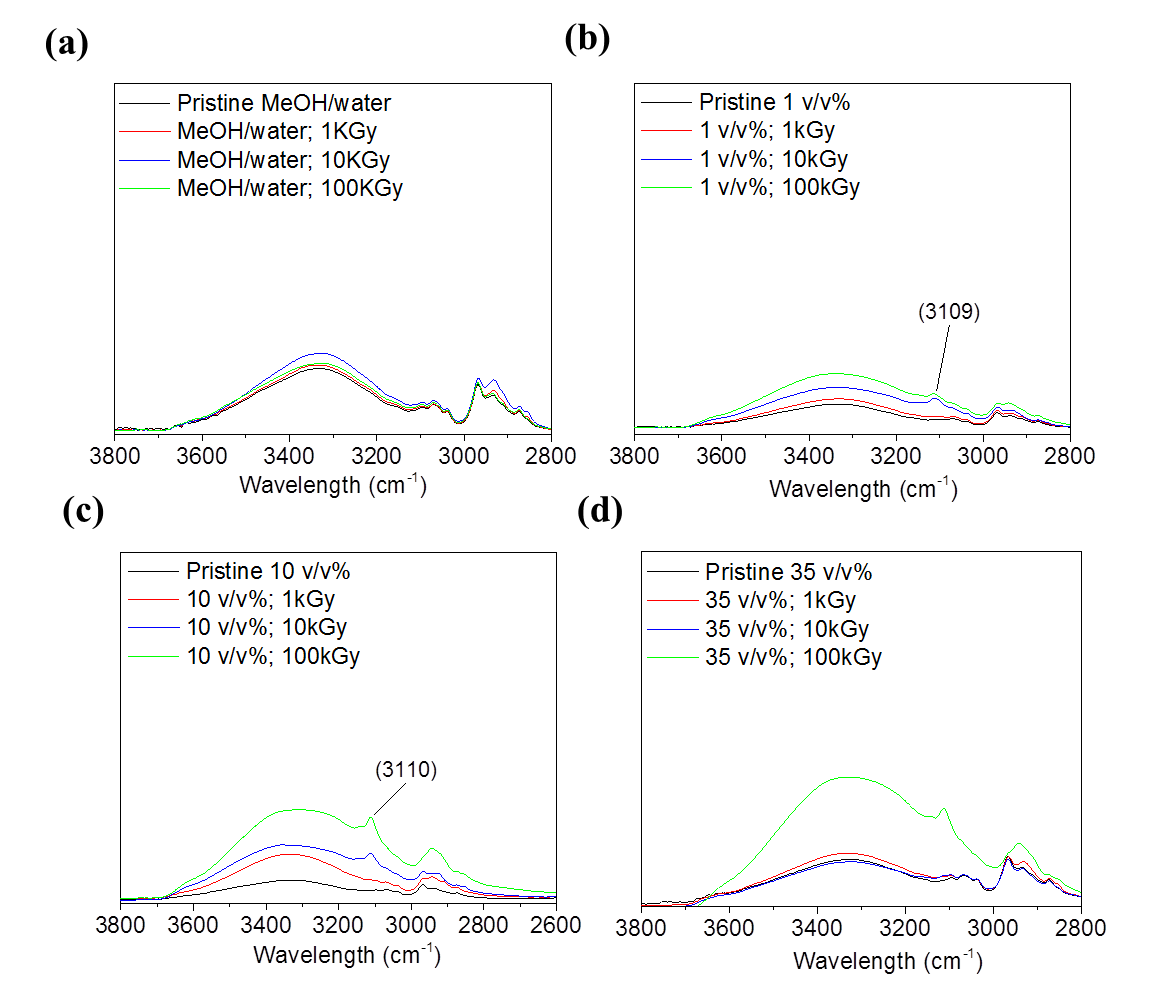


**Figure S7**: FTIR analysis of 3300 cm^-1^ region: a) control membranes in monomer’s solvent (MeOH/water), b) 1% - VIM, c) 10% -VIM and d) 35% - VIM monomer.

The absorbance of the bands corresponding to functional groups in the vicinity of the aromatic amide bands at 1663, 1609 and 1545 cm^-1^ were also enhanced after irradiation grafting (Figure S7). In the same region an extra band at 1649 cm^-1^ was formed and increased with monomer concentration, likely correlated to primary amine pendant groups [19].


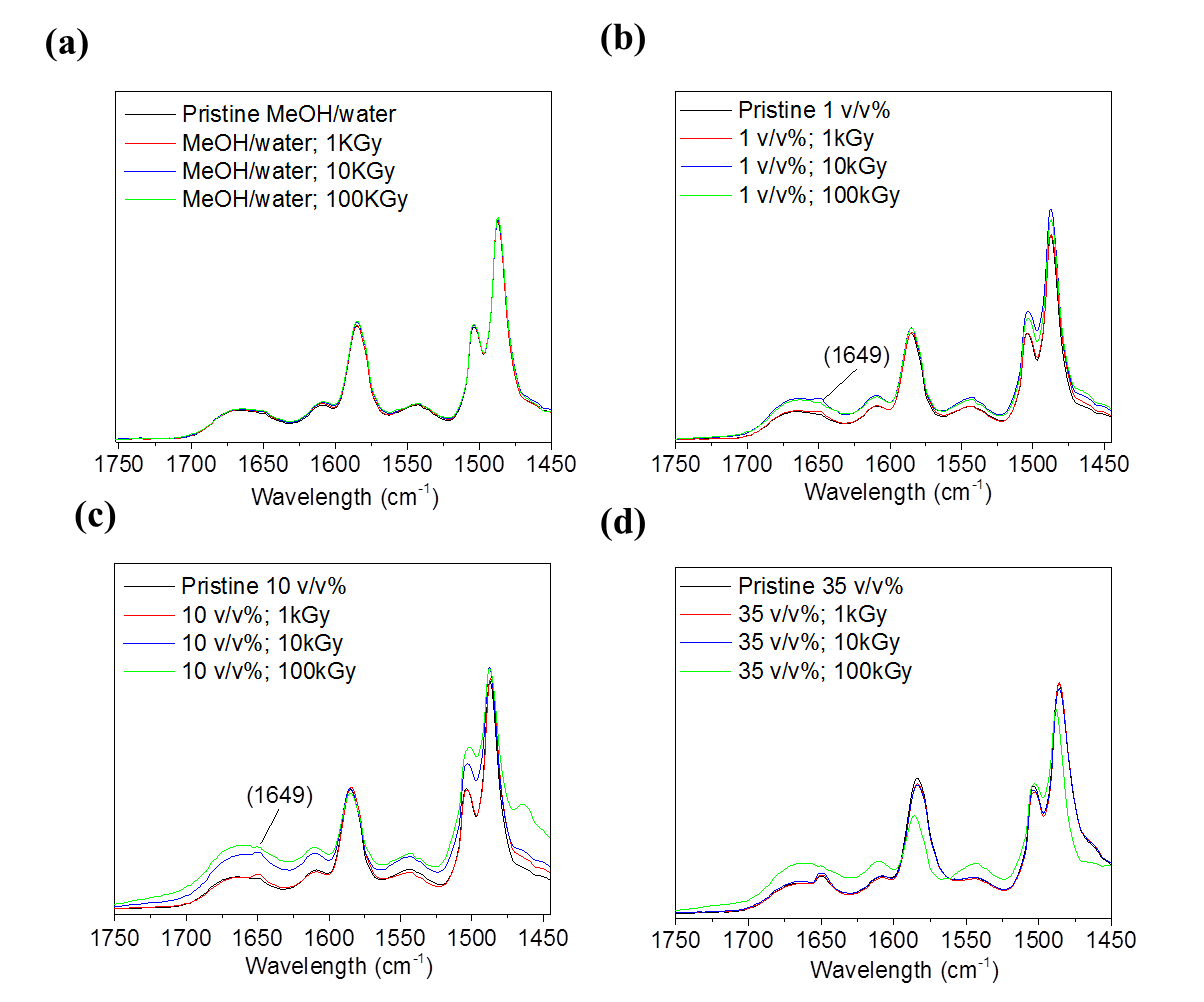


**Figure S_8_:** FTIR analysis from 1750 to 1450 cm^-1^ region: a) control membranes in monomer’s solvent (MeOH/water), b) 1% - VIM, c) 10% -VIM and d) 35% - VIM monomer.

In addition, Figure S9 shows the enhanced band at 918 cm-1, is attributed to C-H out-of–plane bending potentially formed from dissociated vinyl groups from the imidazole ring [20].


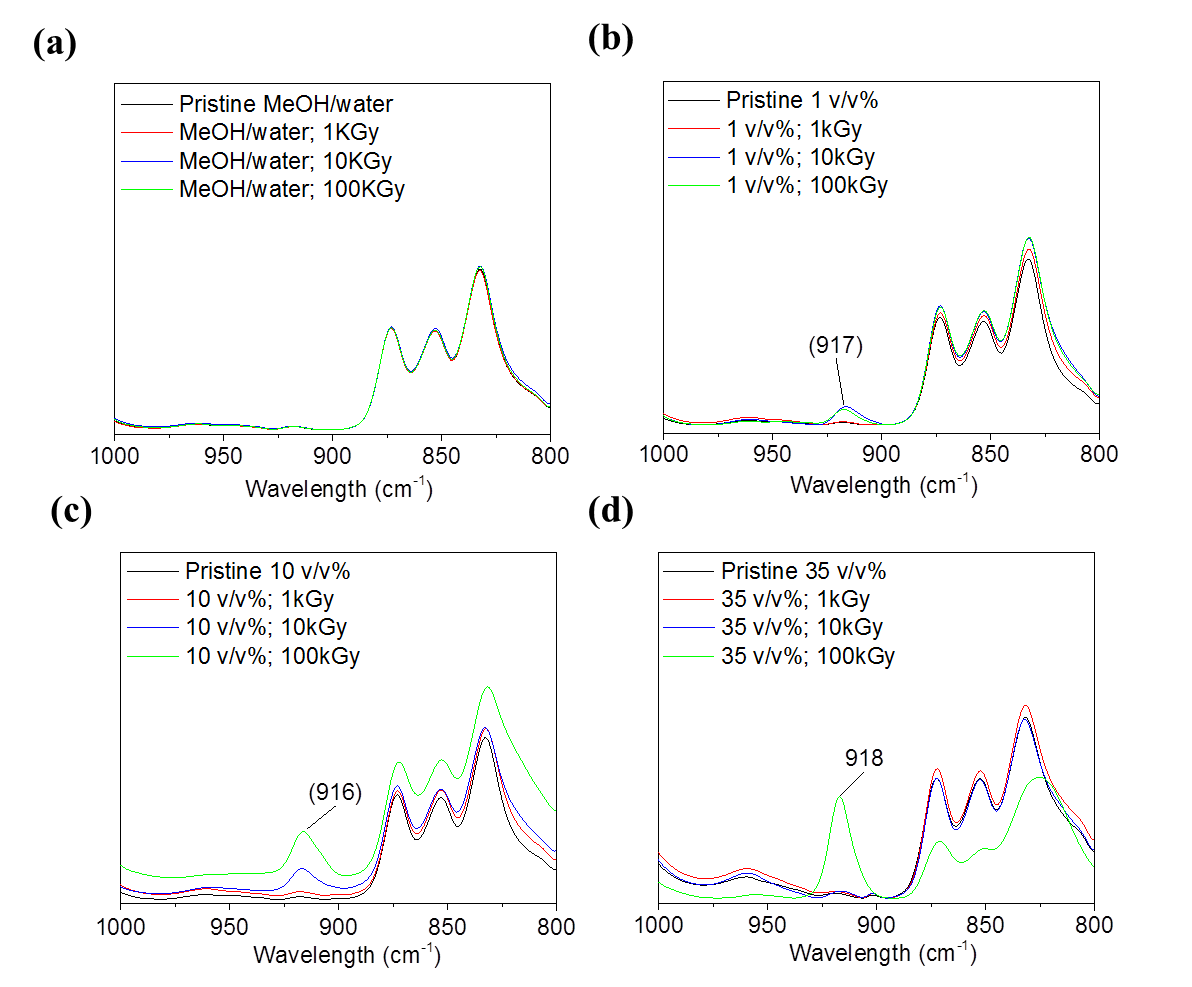


Figure S9: FTIR analysis from 1000 to 800 cm^-1^ region: a) control membranes in monomer’s solvent (MeOH/water), b) 1% - VIM, c) 10% -VIM and d) 35% - VIM monomer.

**
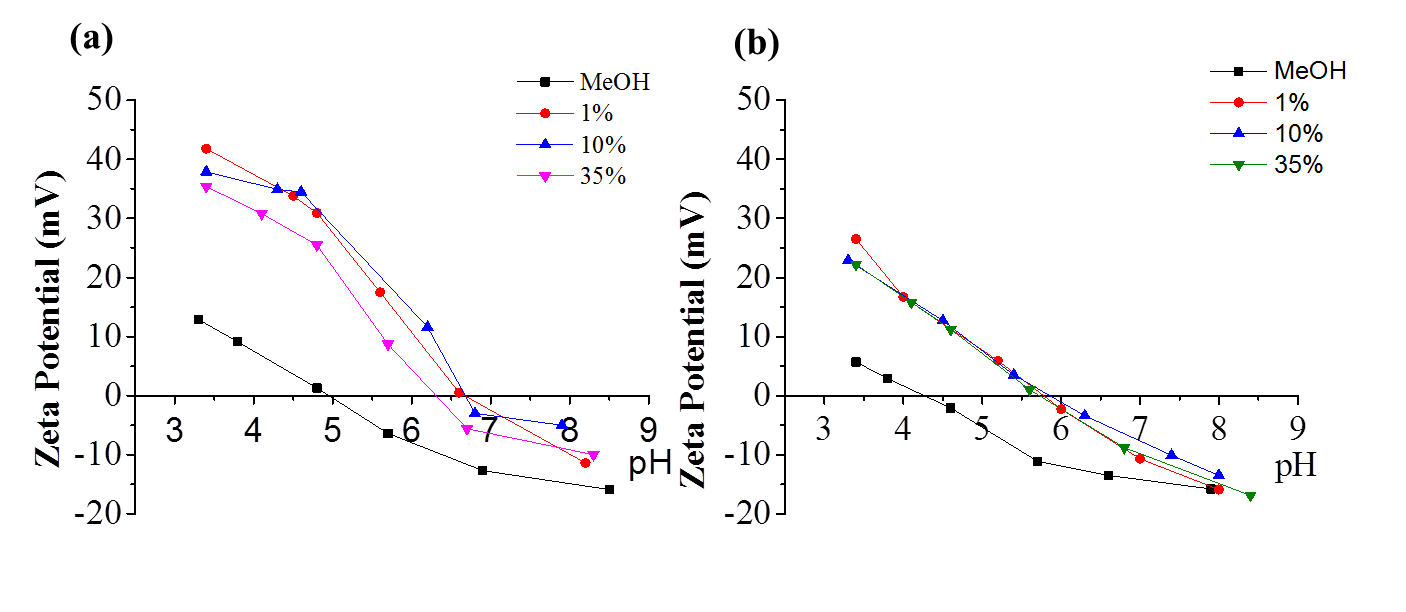
**

**Figure S10:** Streaming potential analyses of irradiation-induced grafted TFC membranes with VIM monomer grafted at 1 kGy total dose with increasing monomer concentration, a) initial performance and b) performance after permeation
